# Solving the Integral Differential Equations with Delayed Argument by Using the DTM Method

**DOI:** 10.3390/s22114124

**Published:** 2022-05-29

**Authors:** Edyta Hetmaniok, Mariusz Pleszczyński, Yasir Khan

**Affiliations:** 1 Department of Mathematics Applications and Methods for Artificial Intelligence, Faculty of Applied Mathematics, Silesian University of Technology, 44-100 Gliwice, Poland; edyta.hetmaniok@polsl.pl; 2 Department of Mathematics, University of Hafr Al Batin, Hafr Al Batin 31991, Saudi Arabia; yasirkhan@uhb.edu.sa

**Keywords:** nonlinear equations, ordinary differential equations, difference equations, integral equations, differential transform method, internet of things, wireless sensor network

## Abstract

Recently, a lot of attention has been paid to the field of research connected with the wireless sensor network and industrial internet of things. The solutions found by theorists are next used in practice in such area as smart industries, smart devices, smart home, smart transportation and the like. Therefore, there is a need to look for some new techniques for solving the problems described by means of the appropriate equations, including differential equations, integral equations and integro-differential equations. The object of interests of this paper is the method dedicated for solving some integro-differential equations with a retarded (delayed) argument. The proposed procedure is based on the Taylor differential transformation which enables to transform the given integro-differential equation into a respective system of algebraic (nonlinear, very often) equations. The described method is efficient and relatively simple to use, however a high degree of generality and complexity of problems, defined by means of the discussed equations, makes impossible to obtain a general form of their solution and enforces an individual approach to each equation, which, however, does not diminish the benefits associated with its use.

## 1. Introduction

In recent times, a dynamically developing field of research is the wireless sensor network and industrial internet of things. Theoretical solutions connected with these topics are next used in practical applications, in such area as smart industries, smart devices, smart home, smart transportation and the like. Therefore, there is a necessity to search for some new techniques for solving the problems described by means of appropriate equations, including differential equations, integral equations and integro-differential equations, in particular with a retarded argument.

Differential equations and integral equations with a retarded argument can find an application in many areas of science, among others, in mechanics, biophysics and control theory [1,2,3,4]. The integro-differential equation with a retarded argument in its full general form is not easy to find in mathematical models expressing the real existing processes, but some specific cases of this equations can be used in description of phenomena with viscoelastic effects [5,6], in the population dynamics, for example in the description of predator-prey models [7], as well as in modeling the problems connected with wireless sensor network and internet of things [8]. Analyzing the subject literature it is quite difficult to find any practical papers concerning the integral and integro-differential equations. The Taylor differential transformation, thanks to its properties, very often simplifies significantly the investigated problem, therefore it has a number of applications in mathematics, engineering and technics. Authors of this paper applied the Taylor differential transformation for solving, among others, the nonlinear ordinary differential equations, variational problems, systems of nonlinear ordinary differential equations, integro-differential equations, Stefan problem, as well as the integral equations [9,10,11]. The discussed transformation is also used by many authors for solving various problems, for example numerous kinds of differential equations (ordinary and partial) and integral equations, there exist also few well known papers devoted to the differential equations with a retarded argument [1,12,13,14,15,16,17,18]. The Taylor differential transformation is popular for a long time, but only recently the methods based on this transformation became useful due to the development of computers and programs enabling to execute the symbolic calculations, for example, in [19] the Authors apply the differential transform method to find the analytic solution to some delay differential equations with the help of the computer algebra system *Mathematica*, whereas in [20] DTM is compared with the other iterative method: the Daftardar-Gejji and Jafari Method in solving the differential equations. In this paper we also apply the *Mathematica* software in version 12.2 [21,22].

In this paper we discuss the integral differential equations with the delayed, or retarded, argument of the following form

(1) $$\begin{array}{cc} f(x,y(x & +\alpha_{0}),y^{\prime }\left(x+\alpha_{1}\right),\ldots ,y^{\left(k\right)}\left(x+\alpha_{k}\right))\\ + & \sum_{i=1}^{n}\int_{x_{1}}^{x_{2}}g_{i}\left(x,y\left(x+\beta_{i}\right)\right)dx+\sum_{i=1}^{m}\int_{x_{0}}^{x}h_{i}\left(t,y\left(t+\gamma_{i}\right)\right)dt=0, \end{array}$$

with conditions

(2) $$y^{\left(i\right)}\left(\varepsilon_{i}\right)=\zeta_{i},\;\;\;i=0,1,\ldots ,k-1,$$

where a≤x≤b, $x+\alpha_{i},x+\beta_{j},t+\gamma_{k}\in \langle a,b\rangle$, 0≤i≤k, 1≤j≤n, 1≤k≤m, $x_{0},x_{1},x_{2}\in \langle a,b\rangle$, whereas the functions *f*, $g_{i}$, i=1,2,…,n, $h_{j}$, j=1,2,…,m, are continuous in set 〈a,b〉×R. Similarly $\varepsilon_{i}\in \langle a,b\rangle$, $\zeta_{i}\in R$, 0≤i≤k−1.

In Equation (1) the functions *f*, *g* and *h* can be nonlinear, whereas the integrals, occurring in this equation, can take the particular forms of integrals appearing in the Fredholm and Volterra integral equations, or they can have such form only with respect to the boundaries of integration, while the integrands g(x) and h(x) can be nonlinear also with respect to the sought function y(x).

Equation (1) will be solved with the aid of the Taylor differential transformation, which, due to its properties, transforms this equation to the respective system of algebraic (nonlinear, very often) equations. The unknown elements in this systems are the coefficients of the Taylor series expanding the sought function. After solving the developed system of equations the approximate solution of the investigated equation can be determined.

Equation (1) in its full form appears rather rarely in the mathematical models describing some physical phenomena. One can find however the specific cases of this equation in the description of phenomena with viscoelastic effects. First papers concerning this problem were developed in the 20th century (by Boltzmann, Maxwell and Kelvin) and were devoted to investigating the creep and recovery phenomena in various materials. This theory evolved in the 20th century thanks to the research on polymers and this development continues even today. For example, paper [5] includes many models describing the viscoelasticity phenomenon, whereas in paper [6] one can find the Laederman model describing the reaction of the fibers creep to the predetermined tensions. This connection is expressed by the relation

$$\varepsilon \left(t\right)=J_{0,\sigma }\sigma \left(t\right)+\int_{0}^{t}J\left(t-\tau \right)\frac{d(g(\sigma (t)))}{dt}d\tau ,$$

where ε denotes the deformation, $J_{0,\sigma }$ means the initial creep compliance, *J* is the creep compliance, σ denotes the tension, and g(σ) is the tension function.

Particular cases of Equation (1) can be also found in the population dynamics, for example in the predator-prey model, described at first by Volterra [7] with the aid of the following system of equations (a similar issue was considered e.g., in work [23])

(3) $$\left\{\begin{array}{c} N_{1}^{\prime }\left(t\right)=\left(b_{1}-a_{1}N_{2}\left(t\right)\right)N_{1}\left(t\right),\\ N_{2}^{\prime }\left(t\right)=\left(a_{2}N_{1}\left(t\right)-b_{2}+\int_{-r}^{0}k\left(s\right)N_{1}\left(t+s\right)ds\right)N_{2}\left(t\right), \end{array}\right.$$

where $N_{1}$ and $N_{2}$ denote the number of predator and prey populations, respectively.

Model (3), modified by Brelot in [24], takes the form

(4) $$\left\{\begin{array}{c} y^{\prime }\left(x\right)=y\left(x\right)\left(r_{1}-a_{11}y\left(x\right)-a_{12}\int_{-\infty }^{x}F\left(x-s\right)z\left(s\right)ds\right),\\ z^{\prime }\left(x\right)=z\left(x\right)\left(a_{21}\int_{-\infty }^{x}G\left(x-s\right)y\left(s\right)ds-r_{2}-a_{22}z\left(x\right)\right), \end{array}\right.$$

where $r_{1}$ denotes the growth rate of the prey population under the assumption of the predators absence, $a_{11}$ is the self-regulation constant of the prey population, $a_{12}$ describes the predation of the prey by predators, $r_{2}$ denotes the death rate of predators under the assumption of the prey absence, $a_{21}$ is the conversion rate of predators, $a_{22}$ describes the intraspecies competition between the predators, functions *F* and *G* are the retardation functions responsible for including the prey and predators history into the model. Specific case of the equation System (4) is solved in Example 5.

Goal of this elaboration is to discuss the specific cases of the integro-differential equations with a retarded argument solved by using the new approach. Deriving the solution formula for the general form of the equation under consideration is impossible, but the discussed examples explain the way how to deal with these problems.

Novelty of this paper is a new approach to the solution of the equation in general form (1), based on the Taylor differential transformation. Contribution of the presented research is the development of an efficient technique for the solution of integro-differential equations with a retarded (delayed) argument with the use of the Taylor differential transformation and showing that the presented solution technique is efficient and relatively simple to use.

In Section 1 we introduce the equations being the object of our interests. Section 2 includes the theoretical description of the Taylor transformation together with its properties, particularly useful in the introduced solution procedure. Some of the properties are proved by example. In Section 3 we explain the details of the solution method and finally in Section 4 we illustrate the theoretical description with five example. The Section 5 contains some comments and conclusions.

## 2. The Taylor Transformation

Let us assume that we consider only such functions of the real variable *x*, defined in some region X⊂R, which can be expanded into the Taylor series within some neighborhood of point α∈X. We call such functions as the originals and denote by the small letters of Latin alphabet, for example *f*, *y*, *u*, *v*, *w*, and so on. Thus, if the function *y* is the original, then the following equality holds

(5) $$y\left(x\right)=\sum_{k=0}^{\infty }\frac{y^{\left(k\right)}\left(\alpha \right)}{k!}{\left(x-\alpha \right)}^{k},$$

where α∈X is the point, in the neighborhood of which the function *y* is expanded into the Taylor series. Each original is assigned to a function $Y_{\alpha }$ of nonnegative integer arguments k=0,1,2,…, according to formula

(6) $$Y_{\alpha }\left(k\right)=\frac{y^{\left(k\right)}\left(\alpha \right)}{k!},\;\;\;k=0,1,2,\ldots$$

The function $Y_{\alpha }$ is called the image of the function *y*, the $T_{\alpha }$–function of the function *y* or the transform of the function *y*, and the discussed transformation is called the Taylor transformation.

The obvious fact is that, by having the $T_{\alpha }$–function $Y_{\alpha }$ one can find, according to Formulas (5) and (6), the corresponding original in the form of its expansion into the Taylor series, that is

(7) $$y\left(x\right)=\sum_{k=0}^{\infty }Y_{\alpha }\left(k\right){\left(x-\alpha \right)}^{k},\;\;\;x,\alpha \in X.$$

Transformation (6), assigning to each original its image, is called the direct transformation. Whereas the transformation (7), assigning the corresponding original to the image, is called the inverse transformation. Connection between these both transformations will be denoted by means of the following symbols:

$$Y_{\alpha }\left(k\right)=T\left[y\left(x\right);k,\alpha \right],$$

for the direct transformation and

$$y\left(x\right)=T^{-1}\left[Y_{\alpha }\left(k\right);x\right],$$

for the inverse transformation, where T and $T^{-1}$ are the symbols of the proper transformations.

In the used notation, for example for the function y(x)=coshx and α=0, we have

$$Y_{0}\left(k\right)=T\left[\cosh x;k,0\right]=T\left[\frac{1}{2}\sum_{k=0}^{\infty }\frac{1+{\left(-1\right)}^{k}}{k!}x^{k};k,0\right]=\frac{1+{\left(-1\right)}^{k}}{2k!},$$

where k=0,1,2,… Whereas in case of the inverse transformation for the above function we get

$$y\left(x\right)=T^{-1}\left[Y_{0}\left(k\right);x\right]=T^{-1}\left[\frac{1+{\left(-1\right)}^{k}}{2k!};x\right]=\frac{1}{2}\sum_{k=0}^{\infty }\frac{1+{\left(-1\right)}^{k}}{k!}x^{k}=\cosh x.$$

The Taylor transformation possesses a number of properties causing that the application of this tool, with the aid of computational platforms giving the possibility to execute the symbolic calculations, like *Mathematica* for example, is quite simple.

In the current elaboration we use the specific case of the Taylor series—the Maclaurin series, therefore α, constantly equal to zero, will be omitted henceforward.

Particularly, the following properties are very useful [1,13,14,25,26]:

(8) $$T\left[x^{n};k\right]=\delta \left(k-n\right)=\left\{\begin{array}{cc} 1, & k=n,\\ 0, & k\neq n, \end{array}\right.$$

(9) $$T\left[e^{ax};k\right]=\frac{a^{k}}{k!},$$

(10) $$T\left[\sin\left(ax\right);k\right]=\frac{a^{k}}{k!}\sin\frac{\pi k}{2},$$

(11) T[c·u(x);k]=c·U(k),

(12) T[u(x)±w(x);k]=U(k)±W(k),

(13) $$T\left[x^{n}\cdot u\left(x\right);k\right]=\left\{\begin{array}{cc} 0, & k=0,1,\ldots ,n-1,\\ U(k-n), & k\geq n, \end{array}\right.$$

(14) $$T\left[\int_{x_{0}}^{x}u\left(t\right)dt;k\right]=\left\{\begin{array}{cc} 0, & k=0,\\ \frac{U(k-1)}{k}, & k\geq 1, \end{array}\right.$$

(15) $$T\left[u\left(x\right)\int_{x_{0}}^{x}w\left(t\right)dt;k\right]=\left\{\begin{array}{cc} 0, & k=0,\\ \sum_{r=0}^{k-1}\frac{U(r)W(k-r-1)}{k-r}, & k\geq 1, \end{array}\right.$$

(16) $$T\left[u\left(x\right)\int_{x_{0}}^{x}v\left(t\right)w\left(t\right)dt;k\right]=\left\{\begin{array}{cc} 0, & k=0,\\ \sum_{i=0}^{k-1}\sum_{j=0}^{k-i-1}\frac{U(i)V(j)W(k-i-j-1)}{k-i}, & k\geq 1, \end{array}\right.$$

where a,c∈R, n∈N∪{0}, and k=0,1,2,…

Besides the “classic” Properties (8)–(14), the Taylor differential transformation possesses a number of properties corresponding to the retarded argument. Some of them are presented below (derivation of these formulas can be found, among others in [1], whereas some of these formulas, like for example Formula (33), are developed by the Authors):

(17) T[y(x+α);k]=∑r=kNrkαr−kY(r),

(18) T[y(x)z(x+α);k]=∑r=0k∑k1=k−rNk1k−rαk1−k+rY(r)Z(k1),

(19) T[y(x+α)z(x+β);k]=∑r=0k∑k1=rN∑k2=k−rNk1rk2k−rαk1−rβk2−k+rY(k1)Z(k2),

(20) T[y(x)z(x)w(x+α);k]=∑r=0k∑r1=0k−r∑k1=k−r−r1Nk1k−r−r1×αk1−k+r+r1Y(r)Z(r1)W(k1),

(21) T[y(x)z(x+α)w(x+β);k]=∑r=0k∑r1=0k−r∑k1=r1N∑k2=k−r−r1Nk1r1×k2k−r−r1αk1−r1βk2−k+r+r1Y(r)Z(k1)W(k2),

(22) T[y′(x+α);k]=(k+1)∑k1=k+1Nk1k+1αk1−k−1Y(k1),

(23) T[y(x)z(n)(x+α);k]=∑k1=0k∑r=k−k1+nN(k−k1+n)!(k−k1)!rk−k1+n×αr−k+k1−nY(k1)Z(r),

(24) T[y(n)(x+α)z(m)(x+β);k]=∑k1=0k∑r1=k1+nN∑r2=k−k1+mN(k1+n)!k1!×(k−k1+m)!(k−k1)!r1k1+nr2k−k1+m×αr1−k1−nβr2−k+k1−mY(r1)Z(r2),

(25) T[y(x)z(n)(x+α)w(m)(x+β);k]=∑r=0k∑k1=0k−r∑r1=k1+nN∑r2=k−r−k1+mN[(k1+n)!k1!(k−r−k1+m)!(k−r−k1)!r1k1+nr2k−r−k1+m×αr1−k1−nβr2−k+k1−mY(r)Z(r1)W(r2)],

(26) T[∫x0xy(t+α)dt;k]=0,k=0,∑k1=k−1Nk1k−1αk1−k+1Y(k1)k,k≥1,

(27) T[∫x0xy(t)z(t+α)dt;k]=0,k=0,∑r=0k−1∑k1=rNk1rY(k1)Z(k−r−1)αk1−rk,k≥1,

(28) T[∫x0xy(t+α)z(t+β)dt;k]=0,k=0,∑r=0k−1∑k1=rN∑k2=k−r−1Nk1rk2k−r−1αk1−rβk2−k+r+1Y(k1)Z(k2)k,k≥1,

(29) T[∫x0xy(t)z(t+α)w(t+β)dt;k]=0,k=0,∑r=0k−1∑r1=0k−r−1∑k1=r1N∑k2=k−r−r1−1Nk1r1k2k−r−r1−1αk1−r1×βk2−k+r+r1+1Y(r)Z(k1)W(k2)k,k≥1,

(30) T[y(x)∫x0xz(t+α)dt;k]=0,k=0,∑r=0k−1∑k1=k−r−1Nk1k−r−1αk1−k+r+1Y(r)Z(k1)k−r,k≥1,

(31) T[y(x)∫x0xz(t)w(t+α)dt;k]=0,k=0,∑r=0k−1∑r1=0k−r−1∑k1=k−r−r1−1Nk1k−r−r1−1αk1−k+r+r1+1×Y(r)Z(r1)W(k1)k−r,k≥1,

(32) T[y(x)∫x0xz(t+α)w(t+β)dt;k]=0,k=0,∑r=0k−1∑r1=0k−r−1∑k1=r1N∑k2=k−r−r1−1Nk1r1k2k−r−r1−1αk1−r1×βk2−k+r+r1+1Y(r)Z(k1)W(k2)k−r,k≥1,

(33) T[∫0xy(t+α)z(t)dt;k]=F(k)=0,k=0,1k·Z(0)(∑r=k−1Nrk−1αr−k+1Y(r)+−∑r=0k−2(r+1)F(r+1)Z(k−1−r)),k≥1,

α,β∈R, n,m∈N, k=0,1,2,…, where N→∞.

As an example we prove the correctness of Formula (33). For this purpose we prove at first some auxiliary identities (in all these theorems we assume that the occurring functions are the originals).

**Lemma**  **1** (see (14))**.** *Let $f\left(x\right)=\int_{0}^{x}y\left(t\right)dt$. Then we have*

$$F\left(k\right)=\left\{\begin{array}{cc} 0, & k=0,\\ \frac{Y(k-1)}{k}, & k\geq 1. \end{array}\right.$$

**Proof.**  Using the expansion of function *y* into the Taylor (Maclaurin) series, we have

$$\begin{array}{cc} f(x) & =\int_{0}^{x}\sum_{k=0}^{\infty }Y\left(k\right)t^{k}dt=\int_{0}^{x}\left(Y\left(0\right)+Y\left(1\right)t+Y\left(2\right)t^{2}+\ldots \right)dt\\ \; & =0+Y\left(0\right)x+\frac{Y(1)}{2}x^{2}+\frac{Y(2)}{3}x^{3}+\ldots +\frac{Y(k-1)}{k}x^{k}+\ldots , \end{array}$$

hence we get the thesis

$$F\left(k\right)=\left\{\begin{array}{cc} 0, & k=0,\\ \frac{Y(k-1)}{k}, & k\geq 1. \end{array}\right.$$

□

**Lemma** **2** (see (17))**.** *Let f(x)=y(x+α). Then we have*

F(k)=∑r=kNrkαr−kY(r),k≥0,N→∞.

**Proof.**  Using the expansion of function *y* into the Taylor series, we have

f(x)=∑k=0∞Y(k)(x+α)k=Y(0)+Y(1)(x+α)+Y(2)(x+α)2+Y(3)(x+α)3+…=Y(0)+αY(1)+α2Y(2)+α3Y(3)+α4Y(4)…+Y(1)+2αY(2)+3α2Y(3)+4α3Y(4)+…x+Y(2)+32αY(3)+42α2Y(4)+52α3Y(5)+…x2+…+kkY(k)+k+1kαY(k+1)+k+2kα2Y(k+2)+…xk+…,

hence we get

F(k)=kkY(k)+k+1kαY(k+1)+k+2kα2Y(k+2)+…==∑r=kNrkαr−kY(r),k≥0,N→∞.

□

**Lemma** **3.** *If $f\left(x\right)=\frac{y(x)}{z(x)}$, z(0)≠0, then*

$$F\left(k\right)=\frac{1}{Z(0)}\left(Y\left(k\right)-\sum_{r=0}^{k-1}F\left(r\right)Z\left(k-r\right)\right).$$

**Proof.**  Let $y\left(x\right)=\sum_{k=0}^{\infty }Y\left(k\right)x^{k}$, $z\left(x\right)=\sum_{k=0}^{\infty }Z\left(k\right)x^{k}$, then we have

(34) $$f\left(x\right)=\frac{y(x)}{z(x)}=\sum_{k=0}^{\infty }F\left(k\right)x^{k},$$

where F(k), k≥0, are unknown. Since the Relation (34) implies that f(x)z(x)=y(x), therefore we have

$$\begin{array}{cc} \; & \left(F\left(0\right)+F\left(1\right)x+F\left(2\right)x^{2}+\ldots \right)\left(Z\left(0\right)+Z\left(1\right)x+Z\left(2\right)x^{2}+\ldots \right)\\ \; & \;\;=Y\left(0\right)+Y\left(1\right)x+Y\left(2\right)x^{2}+\ldots \end{array}$$

and by comparing the coefficients associated to the respective powers of *x*, we obtain successively

$$F\left(0\right)Z\left(0\right)=Y\left(0\right)\Rightarrow F\left(0\right)=\frac{Y(0)}{Z(0)},$$

$$F\left(0\right)Z\left(1\right)+F\left(1\right)Z\left(0\right)=Y\left(1\right)\Rightarrow F\left(1\right)=\frac{Y(1)-F(0)Y(1)}{Z(0)},$$

$$F\left(0\right)Z\left(2\right)+F\left(1\right)Z\left(1\right)+F\left(2\right)Z\left(0\right)=Y\left(2\right)\Rightarrow F\left(2\right)=\frac{Y\left(2\right)-\left(F(0)Z(2)+F(1)Z(1)\right)}{Z(0)},\ldots ,$$

F(0)Z(k)+F(1)Z(k−1)+F(2)Z(k−2)+…+F(k−1)Z(1)+F(k)Z(0)=Y(k),

hence we get

$$\begin{array}{cc} F(k) & =\frac{Y\left(k\right)-\left(F(0)Z(k)+F(1)Z(k-1)+F(2)Z(k-2)+\ldots +F(k-1)Z(1)\right)}{Z(0)}\\ \; & =\frac{1}{Z(0)}\left(Y\left(k\right)-\sum_{r=0}^{k-1}F\left(r\right)Z\left(k-r\right)\right),\;\;k\geq 1. \end{array}$$

Since for k=0 the last sum is “empty”, so it can be considered that the obtained formula holds true for all integers k≥0. Thus we have

$$F\left(k\right)=\frac{1}{Z(0)}\left(Y\left(k\right)-\sum_{r=0}^{k-1}F\left(r\right)Z\left(k-r\right)\right),\;\;k\geq 0,$$

which is the thesis of the lemma. □

**Theorem** **1** (see (33))**.** *Let $f\left(x\right)=\int_{0}^{x}\frac{y(t+\alpha )}{z(t)}dt$, z(0)≠0. Then F(0)=0 and for k≥1 we have*

F(k)=1k·Z(0)∑r=k−1Nrk−1αr−k+1Y(r)−∑r=0k−2(r+1)F(r+1)Z(k−1−r).

**Proof.**  Applying Lemmas 2 and 3 we can write that if $g\left(x\right)=\frac{y(x+\alpha )}{z(x)}$, z(0)≠0, then

$$G\left(k\right)=\frac{1}{Z(0)}\left(Y_{\alpha }\left(k\right)-\sum_{r=0}^{k-1}G\left(r\right)Z\left(k-r\right)\right),$$

where $Y_{\alpha }\left(k\right)$ is the *T*-function of function y(x+α), that is (see Lemma 2):

Yα(k)=∑r=kNrkαr−kY(r),k≥0,N→∞.

Thus we have

G(k)=1Z(0)∑r=kNrkαr−kY(r)−∑r=0k−1G(r)Z(k−r),k≥0,N→∞.

Now, by applying Lemma 1 we have $f\left(x\right)=\int_{0}^{x}g\left(t\right)dt$, hence we get F(0)=0 and for k≥1 we have

F(k)=1Z(0)1k∑r=k−1Nrk−1αr−(k−1)Y(r)−∑r=0k−1−1Hk,r.

On should pay a special attention to the sum of elements $H_{k,r}$. We cannot simply substitute there the expression k−1 in place of *k*, because the coefficients *F* occur in this sum. Referring to the indices changed in this way we came across the negative indices. Such indices suppose to be properly retarded and the changed value, resulting from this retardation, must be regulated with the aid of appropriate multipliers. Hence we finally get for k≥1:

F(k)=1kZ(0)∑r=k−1Nrk−1αr−(k−1)Y(r)−∑r=0k−2(r+1)F(r+1)Z(k−1−r),

which ends the proof of theorem. □

## 3. Method of Solution

Besides the properties, listed above, for solving the equations of considered kind we will also use the initial Conditions (2). To use them efficiently, one should notice that

$$y\left(x\right)=\sum_{k=0}^{\infty }\frac{y^{\left(k\right)}\left(x_{0}\right)}{k!}{\left(x-x_{0}\right)}^{k}\Rightarrow y^{\left(n\right)}\left(x\right)=\sum_{k=0}^{\infty }\frac{y^{\left(k+n\right)}\left(x_{0}\right)}{k!}{\left(x-x_{0}\right)}^{k},$$

or else

$$y\left(x\right)=\sum_{k=0}^{\infty }Y\left(k\right){\left(x-x_{0}\right)}^{k}\Rightarrow y^{\left(n\right)}\left(x\right)=\sum_{k=0}^{\infty }\frac{(k+n)!}{k!}Y\left(k+n\right){\left(x-x_{0}\right)}^{k},$$

hence, if we take $x_{0}=0$, we get

(35) $$y^{\left(n\right)}\left(\beta \right)=\gamma \Leftrightarrow \sum_{k=0}^{\infty }\frac{(k+n)!}{k!}Y\left(k+n\right)\beta^{k}=\gamma .$$

Let us observe that in Formula (27), and in the similar formulas as well, the lower limit of integration is equal to the point, around which the given function is expanded into the Taylor series. According to the agreement, mentioned before, in this paper we take $x_{0}=0$. If the case arises that in Equation (1) the lower limit of the Volterra type integral will be different than zero, then it is enough to notice, that if (independently whether a<0, a=0, or a>0):

$$u\left(x\right)=\int_{a}^{x}h\left(t,y\left(t+\beta \right)\right)dt=\int_{a}^{0}h\left(t,y\left(t+\beta \right)\right)dt+\int_{0}^{x}h\left(t,y\left(t+\beta \right)\right)dt=\lambda +v\left(x\right),$$

where

$$R∋\lambda =\int_{a}^{0}h\left(t,y\left(t+\beta \right)\right)dt,$$

$$v\left(x\right)=\int_{0}^{x}h\left(t,y\left(t+\beta \right)\right)dt,$$

and

$$T\left[v\left(x\right);k\right]=\left\{\begin{array}{c} 0,\;\;k=0,\\ V(k),\;k\geq 1, \end{array}\right.$$

then

(36) $$T\left[u\left(x\right);k\right]=\left\{\begin{array}{c} \lambda ,\;\;k=0,\\ V(k),\;k\geq 1. \end{array}\right.$$

Obviously, in Formulas (17)–(33) we cannot take the infinite sums. Instead, the summation will be executed until the fixed N∈N. We obtain then N+1 equations with unknowns: Y(0), Y(1), …, Y(N). After solving this system of equations we receive the following approximate solution

$$y_{N}\left(x\right)=\sum_{k=0}^{N}Y\left(k\right)x^{k}.$$

## 4. Examples

**Example** **1.** *We consider the equation*

(37) $$\begin{array}{cc} y^{\prime }\left(x-1\right)y^{\prime }\left(x+1\right) & +9xy\left(x\right)-\frac{21}{20}\int_{-1}^{1}y\left(x\right)y\left(x-1\right)dx\\ \; & +4\int_{0}^{x}\left(y\left(t\right)-y\left(t-1\right)\right)dt=4x\left(3-x^{2}\right), \end{array}$$

*with condition*

(38) y(−2)=0,

*the exact solution of which is given by function $y\left(x\right)=x\left(4-x^{2}\right)$.*

Equation (37), under assumption $\frac{21}{20}\int_{-1}^{1}y\left(x\right)y\left(x-1\right)dx=\lambda \in R$ and after applying, among others, the Properties (8), (11), (12), (13), (14), (24) and (26), transforms to the form

(39) ∑r=0k∑k1=r+1N∑k2=k−r+1N[(r+1)(k−r+1)k1r+1k2k−r+1(−1)k1−r−1×Y(k1)Y(k2)]+0,k=0,9Y(k−1)+4Y(k−1)k,k≥1−λδ(k−0)−40,k=0,∑k1=k−1Nk1k−1(−1)k1−k+1Y(k1)k=12δ(k−1)−4δ(k−3)

with the following equation included, on the grounds of initial Condition (38) and Formula (35):

(40) $$\sum_{i=0}^{N}Y\left(i\right){\left(-2\right)}^{i}=0.$$

Taking N=3, Equation (39) leads to a system of equations of the form

(41) $$\left\{\begin{array}{c} -\lambda +{\left(Y\left(1\right)\right)}^{2}-4{\left(Y\left(2\right)\right)}^{2}+6Y\left(1\right)Y\left(3\right)+9{\left(Y\left(3\right)\right)}^{2}=0,\\ 9Y(0)+4(Y(1)(1+Y(2))+Y(3)-Y(2)(1+3Y(3)))=12,\\ 4Y\left(2\right)+4{\left(Y\left(2\right)\right)}^{2}-6Y\left(3\right)\left(1+3Y\left(3\right)\right)+3Y\left(1\right)\left(3+2Y\left(3\right)\right)=0,\\ 4Y(3)+3Y(2)(3+4Y(3))=-4\\ Y(0)-2Y(1)+4Y(2)-8Y(3)=0. \end{array}\right.$$

Solving the system of Equations (41), we get three groups of solutions. After the appropriate verification (we assess the quality of the obtained approximate solution by substituting it into Equation (37)), it turns out that the best solution is Y(0)=0, Y(1)=4, Y(2)=0, Y(3)=−1 and λ=1, that is the function $y_{3}\left(x\right)=4x-x^{3}$, which is the exact solution. For N>3 we obtain the same values λ, Y(i), 0≤i≤3 and Y(i)=0, i≥4, that is the same exact solution. It is worth noting that we did not need to use here the definition of λ.

**Example** **2.** *We consider the equation*

(42) $$\begin{array}{cc} {\left(y^{\prime \prime }\left(x-\frac{\pi }{2}\right)\right)}^{2} & -2\pi y^{\prime }\left(x-\frac{\pi }{2}\right)+\frac{1}{2}\int_{0}^{x}{\left(y\left(x+\frac{\pi }{2}\right)-y\left(x-\frac{\pi }{2}\right)\right)}^{2}dt\\ \; & =\frac{1}{2}\left(4+\pi^{2}\right)x+\cos2x-\sin2x, \end{array}$$

*for $-\frac{\pi }{2}\leq x\leq \frac{\pi }{2}$, with conditions*

(43) $$y\left(-\frac{\pi }{4}\right)=-\frac{\pi }{4},\;\;y^{\prime }\left(0\right)=0,$$

*the exact solution of which is given by function y(x)=x−sinx−cosx.*

Equation (42), in result of applying, among others, the Properties (8), (10)–(12), (22)–(26) and (28), and using the initial Condition (43) (Y(1)=0), develops into the form

(44) ∑r=0k∑k1=r+2N∑k2=k−r+2N[(r+1)(r+2)(k−r+1)(k−r+2)k1r+2k2k−r+2×−π2k1+k2−k−4Y(k1)Y(k2)]−2π(k+1)∑k1=k+1Nk1k+1−π2k1−k−1Y(k1)+120,k=0,∑r=0k−1∑k1=rN∑k2=k−r−1Nk1rk2k−r−1π2k1+k2−k+1Y(k1)Y(k2)k,k≥1−0,k=0,∑r=0k−1∑k1=rN∑k2=k−r−1Nk1rk2k−r−1π2k1−r−π2k2−k+r+1×Y(k1)Y(k2)k,k≥1+120,k=0,∑r=0k−1∑k1=rN∑k2=k−r−1Nk1rk2k−r−1−π2k1+k2−k+1Y(k1)Y(k2)k,k≥1=coskπ2−sinkπ2k!2k+12(4+π2)δ(k−1),

with the following equation included, basing on the initial Condition (43) and Formula (35):

(45) $$\sum_{i=0}^{N}Y\left(i\right){\left(-\frac{\pi }{4}\right)}^{i}=-\frac{\pi }{4}.$$

The unknowns of this system are Y(0), Y(2),Y(3),…,Y(N). Taking N=5 in Relations (44)–(45) we get the system of equations, the solution if which are Y(0)=−0.9759, Y(2)=0.4389, Y(3)=0.1656, Y(4)=−0.0071, Y(5)=−0.0091, which gives

$$y_{5}\left(x\right)=-0.9759+0.4389x^{2}+0.1656x^{3}-0.0071x^{4}-0.0091x^{5},$$

whereas by taking N=9 we obtain the approximate solution $y_{9}\left(x\right)=\sum_{i=0}^{9}Y\left(i\right)x^{i}:$

$$\begin{array}{cc} y_{9}\left(x\right)= & -0.9953+0.4923x^{2}+0.177x^{3}-0.0296x^{4}-0.0128x^{5}-0.0021x^{6}\\ \; & +0.0006x^{7}+0.0002x^{8}+0.0000x^{9}. \end{array}$$

**Figure 1:**
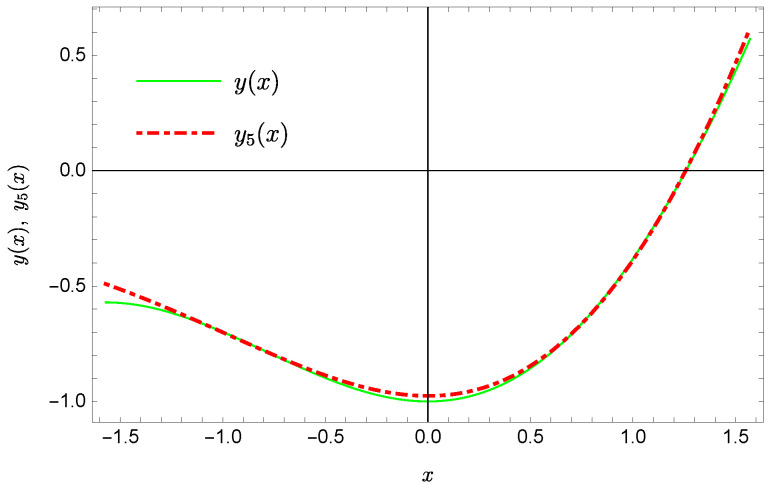
Exact solution and the approximate solution $y_{5}\left(x\right)$.

**Figure 2:**
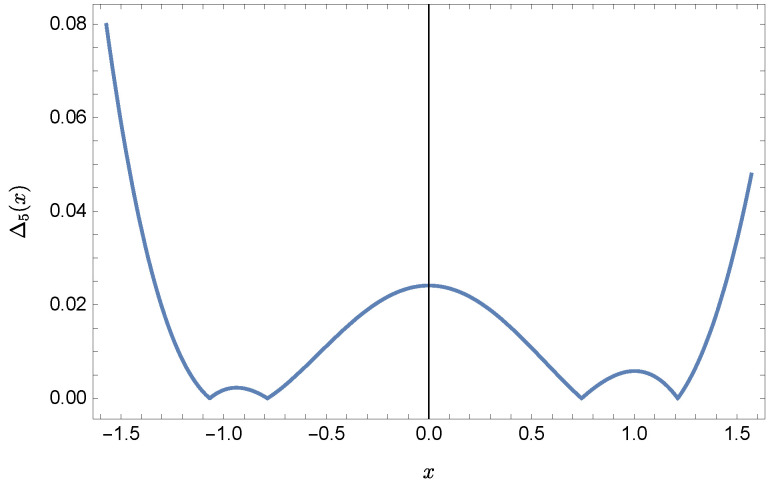
Error $\Delta_{5}$ of approximation $y_{5}\left(x\right)$.

**Figure 3:**
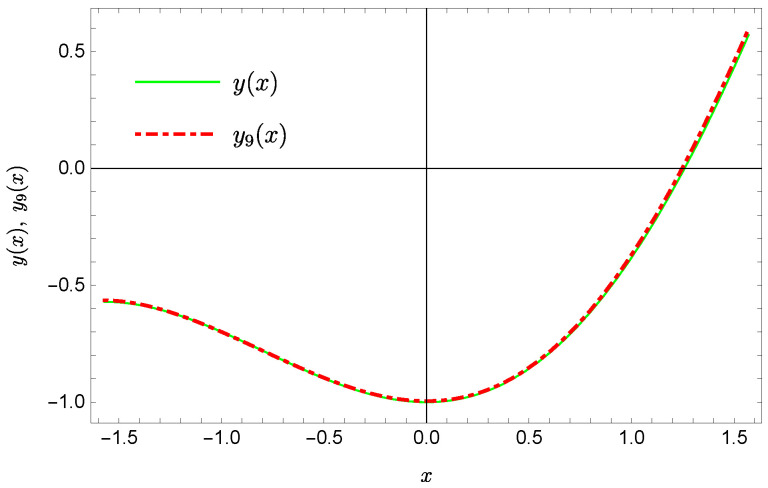
Exact solution and the approximate solution $y_{9}\left(x\right)$.

**Figure 4:**
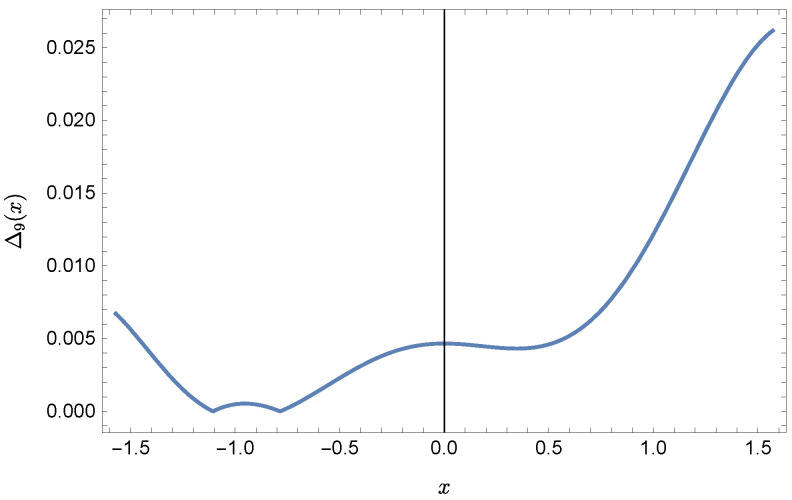
Error $\Delta_{9}$ of approximation $y_{9}\left(x\right)$.

Plots of the received solutions, together with the plots of absolute errors $\Delta_{N}\left(x\right)$ of these solutions

$$\Delta_{N}\left(x\right)=\left|y\left(x\right)-y_{N}\left(x\right)\right|,$$

are presented in Figure 1, Figure 2, Figure 3 and Figure 4. In Figure 1 and Figure 3 the solid green line represents the exact solution, whereas the dashed red line marks the approximate solution. The errors Δ are displayed in Figure 2 and Figure 4.

**Example** **3.** *Let us consider the equation*

(46) $$\begin{array}{cc} e^{-2x}y^{\prime }\left(x-1\right)y^{\prime \prime }\left(x+1\right) & -\frac{15}{4}\int_{-1}^{1}\left(x+1\right)y\left(x+1\right)dx\\ \; & +\frac{6}{e}\int_{-1}^{x}\left(t+2\right)e^{-t}y\left(t+1\right)dt=x\left(2x^{2}+4x-13\right), \end{array}$$

*for −2≤x≤2, with conditions*

(47) $$y\left(0\right)=-2,\;\;y^{\prime }\left(-1\right)=-\frac{2}{e},$$

*the exact solution of which is defined by function $y\left(x\right)=\left(x-2\right)e^{x}$.*

Equation (46), under assumption

$$\frac{15}{4}\int_{-1}^{1}\left(x+1\right)y\left(x+1\right)dx=\lambda_{1}\in R$$

and after applying the Property (36), as well as under assumption

$$\frac{6}{e}\int_{-1}^{0}\left(x+2\right)e^{-x}y\left(x+1\right)dx=\lambda_{2}\in R$$

and after applying, among others, the Properties (8), (9), (11), (12), (13), (25) and (27), transforms into the form

(48) ∑r=0k∑r1=0k−r∑k1=r1+1N∑k2=k−r−r1+2N[(r1+1)(k−r−r1+1)(k−r−r1+2)×k1r1+1k2k−r−r1+2(−1)k1−r1−1(−2)rr!Y(k1)Y(k2)]+6e0,k=0,∑r=0k−1∑r1=0k−r−1∑k1=k−r−r1−1Nk1k−r−r1−1×(δ(r−1)+2δ(r−0))(−1)r1Y(k1)k·r1!,k≥1+(λ2−λ1)δ(k−0)=2δ(k−3)+4δ(k−2)−13δ(k−1),

where Y(0)=−2 basing on Condition (47), with the following equation included, on the grounds of initial Condition (47) and Formula (35):

(49) $$\sum_{i=0}^{N-1}\left(i+1\right)Y\left(i+1\right){\left(-1\right)}^{i}=-\frac{2}{e}$$

and the definitions of constants $\lambda_{1}$ and $\lambda_{2}$ given below

(50) $$\begin{array}{cc} \frac{15}{4} & \int_{-1}^{1}\left(x+1\right)\sum_{i=0}^{N}Y\left(i\right){\left(x+1\right)}^{i}dx=\lambda_{1},\\ \frac{6}{e} & \int_{-1}^{0}\left(x+2\right)e^{-x}\sum_{i=0}^{N}Y\left(i\right){\left(x+1\right)}^{i}dx=\lambda_{2}. \end{array}$$

Taking the fixed value of *N* in Equations (48)–(50) we get the system of equations with unknowns Y(1), Y(2),…,Y(N). So, taking N=6, we receive in result the following approximate solution

(51) $$y_{6}\left(x\right)=-2-0.9159x-0.037x^{2}+0.1063x^{3}+0.0901x^{4}+0.0394x^{5}+0.0082x^{6}$$

with $\lambda_{1}=-14.8719$ and $\lambda_{2}=-12.9006$ (their exact values are $\lambda_{1}=-15$, $\lambda_{2}=-13$). Whereas for N=9 we obtain the following approximate solution

(52) $$\begin{array}{cc} y_{9}\left(x\right)=- & \;2-1.0049x+0.002x^{2}+0.1701x^{3}+0.083x^{4}+0.0242x^{5}\\ + & \;0.0054x^{6}+0.001x^{7}+0.0002x^{8}+0.00002x^{9} \end{array}$$

with $\lambda_{1}=-15.0077$ and $\lambda_{2}=-13.006$.

**Figure 5:**
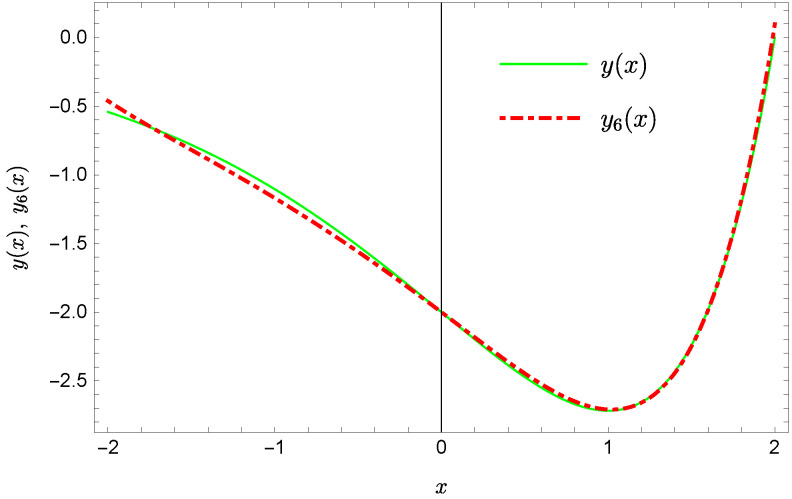
Exact solution and the approximate solution $y_{6}\left(x\right)$.

**Figure 6:**
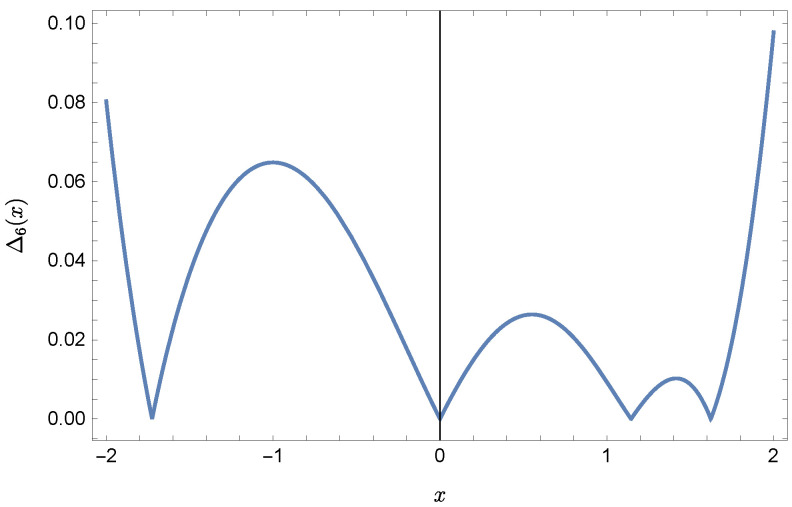
Error $\Delta_{6}$ of approximation $y_{6}\left(x\right)$.

**Figure 7:**
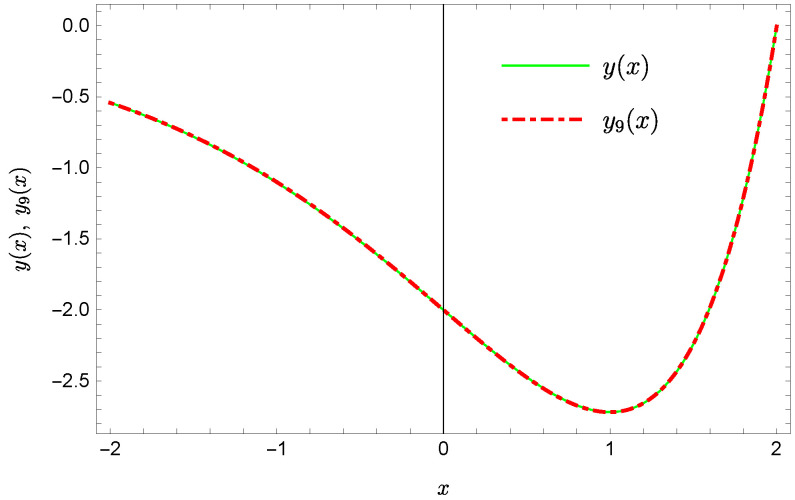
Exact solution and the approximate solution $y_{9}\left(x\right)$.

**Figure 8:**
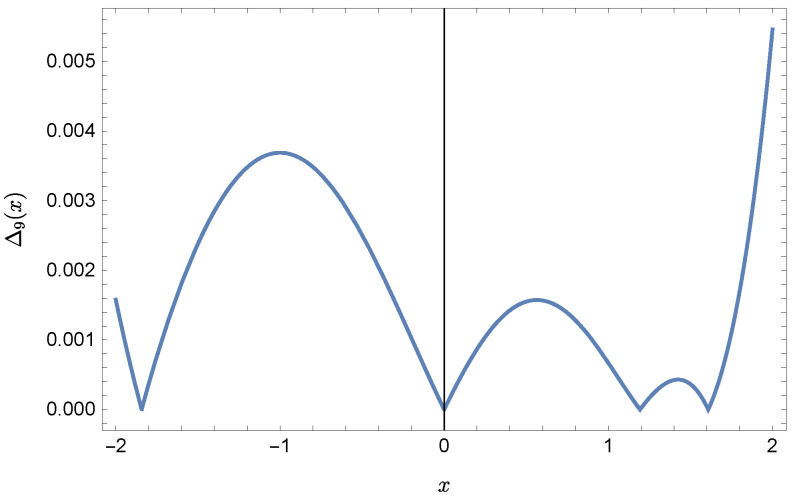
Error $\Delta_{9}$ of approximation $y_{9}\left(x\right)$.

Plots of the approximate Solutions (51) and (52), the exact solution and the absolute errors $\Delta_{N}\left(x\right)$ of these approximations are displayed in Figure 5, Figure 6, Figure 7 and Figure 8 (the notation of figures is the same as previously).

**Example** **4.** *Now we take into consideration the following equation*

(53) $$\begin{array}{cc} (e^{-x} & +y^{\prime \prime }\left(x-1\right))\left(e^{-x}-y^{\prime \prime }\left(x+1\right)\right)+2\int_{0}^{1}y\left(x\right)y\left(x-1\right)dx\\ \; & +e\int_{0}^{x}\frac{1-y(t-1)}{1-y(t)}dt=2+x-2\sinh2x, \end{array}$$

*for −2≤x≤2, with conditions*

(54) $$y\left(0\right)=0,\;\;y^{\prime }\left(0\right)=-1,$$

*the exact solution of which is described by function $y\left(x\right)=1-e^{x}$.*

Equation (53), in result of applying the Property (36), that is assuming that

$$2\int_{0}^{1}y\left(x\right)y\left(x-1\right)dx=\lambda \in R$$

and in result of using, among others, the Properties (8), (9), (11), (12), (23), (24) and (33), develops into the form

(55) ∑r=0k∑k1=k−r+2N(k−r+2)!(k−r)!k1k−r+2(−1)k1−k+r−2(−1)rr!Y(k1)−∑r=0k∑k1=k−r+2N(k−r+2)!(k−r)!k1k−r+2(−1)rr!Y(k1)−∑r=0k∑k1=r+2N∑k2=k−r+2N[(r+2)!r!(k−r+2)!(k−r)!k1r+2k2k−r+2×(−1)k1−r−2Y(k1)Y(k2)]+λδ(k−0)+eF(k)=2δ(k−0)+δ(k−1)−2kk!,

where

F(k)=0,k=0,1k(1−Y(0))(∑k1=k−1Nk1k−1(−1)k1−k+1(δ(k1−0)−Y(k1))−∑r=0k−2(r+1)F(r+1)(δ(k−r−1−0)−Y(k−1−r))),k≥1,

where Y(0)=0, Y(1)=−1 basing on Condition (54), with the following definition of constant λ included

(56) $$2\int_{0}^{1}\left(\sum_{i=0}^{N}Y\left(i\right)x^{i}\right)\left(\sum_{i=0}^{N}Y\left(i\right){\left(x-1\right)}^{i}\right)dx=\lambda .$$

Assuming in (55) and (56) the fixed value of *N* we get the system of equations with unknowns Y(2), Y(3),…,Y(N). So, value N=5 leads to the approximate solution

(57) $$y_{5}\left(x\right)=-x-0.5043x^{2}-0.1791x^{3}-0.0503x^{4}-0.0075x^{5}$$

with λ=−0.3524 (the exact value is $\lambda =2+\frac{1}{e}-e\approx -0.3504$). Whereas for N=9 we obtain the approximate solution of the form

(58) $$\begin{array}{cc} y_{9}\left(x\right)=- & \;x-0.4994x^{2}-0.1662x^{3}-0.0417x^{4}-0.0085x^{5}\\ - & \;0.0014x^{6}-0.0002x^{7}-0.00003x^{8}-3\cdot 10^{-6}x^{9} \end{array}$$

with λ=−0.350359.

**Figure 9:**
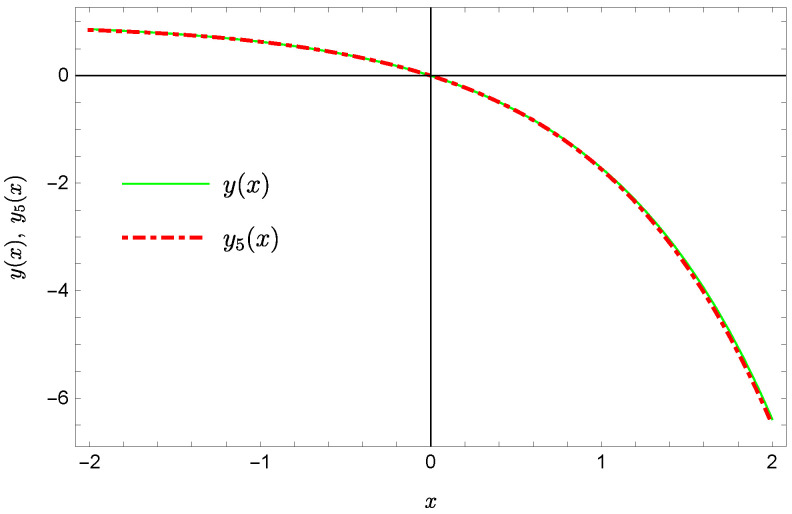
Exact solution and the approximate solution $y_{5}\left(x\right)$.

**Figure 10:**
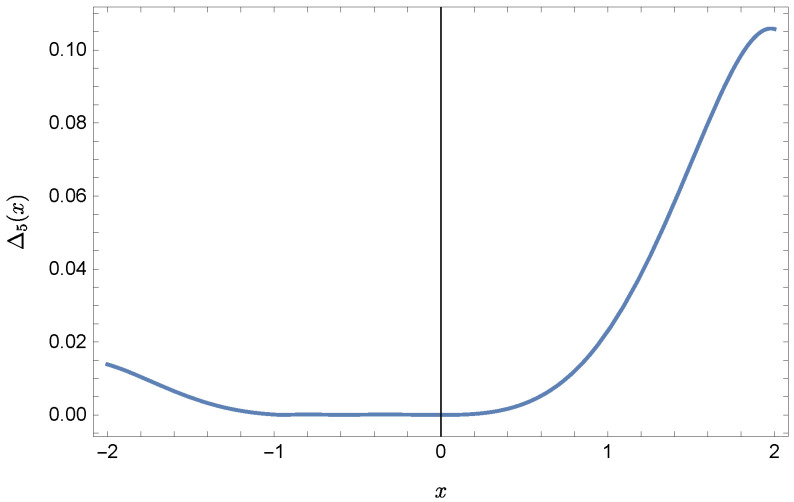
Error $\Delta_{5}$ of approximation $y_{5}\left(x\right)$.

**Figure 11:**
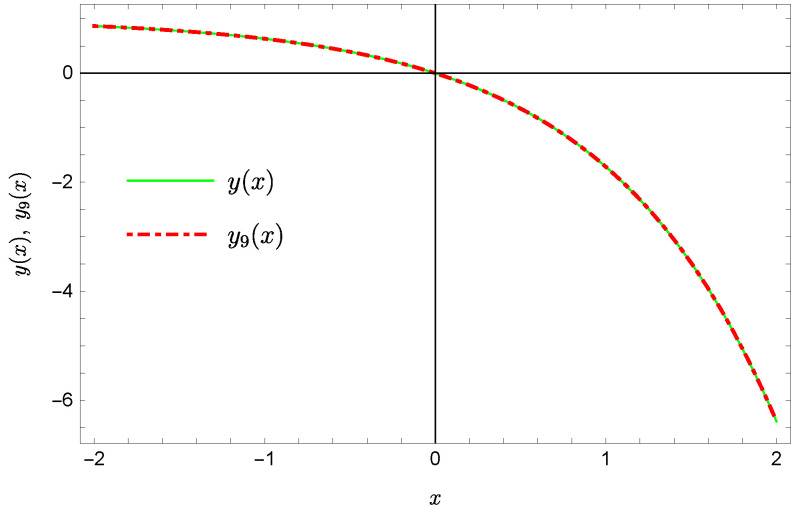
Exact solution and the approximate solution $y_{9}\left(x\right)$.

**Figure 12:**
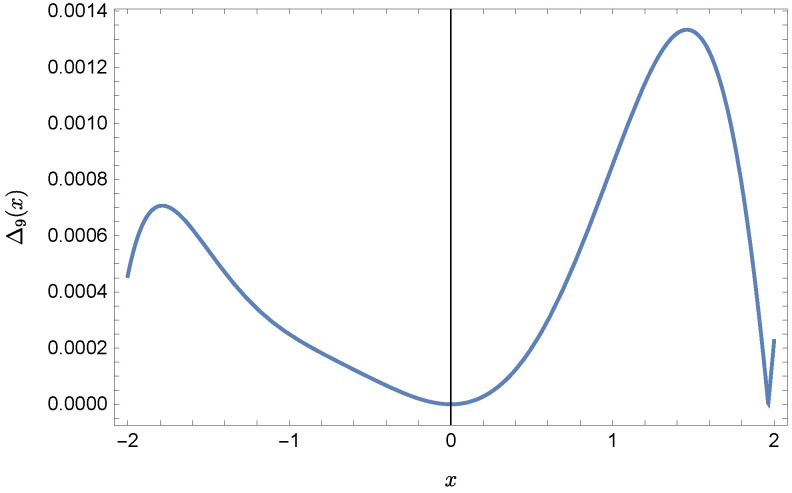
Error $\Delta_{9}$ of approximation $y_{9}\left(x\right)$.

Plots of the approximate Solutions (57) and (58), the exact solution and the absolute errors $\Delta_{N}\left(x\right)$ of these approximations are displayed in Figure 9, Figure 10, Figure 11 and Figure 12 (the previous notation of figures is kept).

**Example** **5.** *Let us solve the specific case of the system of Equation (4), in which we take: $r_{1}=a_{11}=a_{12}=1$, $a_{21}=0$, $r_{2}=1$, $a_{22}=-1$, $F\left(x\right)=e^{x}$. Then the system takes the form*

(59) $$\left\{\begin{array}{c} y^{\prime }\left(x\right)=y\left(x\right)\left(1-y\left(x\right)-\int_{0}^{x}e^{x-s}z\left(s\right)ds\right),\\ z^{\prime }\left(x\right)=z\left(x\right)\left(z\left(x\right)-1\right), \end{array}\right.$$

*for 0≤x≤2, with conditions*

(60) $$y\left(0\right)=-\frac{1}{2},\;\;z\left(0\right)=1,$$

*the exact solution of which is given by functions $y\left(x\right)=-\frac{e^{2x}}{1+e^{x}}$ and z(x)=1.*

System of Equations (59), after applying the properties of the Taylor transformation (see (16) where $u\left(x\right)=y\left(x\right)e^{x}$), can be written in the following form

(61) $$\begin{array}{cc} \left(k+1)Y(k+1\right) & =Y\left(k\right)-\sum_{r=0}^{k}Y\left(r\right)Y\left(k-r\right)\\ \; & -\sum_{i=0}^{k-1}\sum_{j=0}^{k-i-1}\sum_{r=0}^{i}\frac{Y\left(r\right)Z\left(k-i-j-1\right){\left(-1\right)}^{j}}{(k-i)(i-r)!j!},\\ \left(k+1)Z(k+1\right) & =\sum_{r=0}^{k}Z\left(r\right)Z\left(k-r\right)-Z\left(k\right). \end{array}$$

Using the Conditions (60) and taking in Relations (61) the successive values k≥0, we get in turn

— for k=0: $\left\{\begin{array}{c} Y\left(1\right)=-\frac{3}{4},\\ Z(1)=0; \end{array}\right.$
— for k=1: $\left\{\begin{array}{c} 2Y(2)=-1,\\ 2Z(2)=0, \end{array}\right.$ hence we get $\left\{\begin{array}{c} Y\left(2\right)=-\frac{1}{2},\\ Z(2)=0; \end{array}\right.$
— for k=2: $\left\{\begin{array}{c} 3Y\left(2\right)=-\frac{9}{16},\\ 3Z(2)=0, \end{array}\right.$ hence we get $\left\{\begin{array}{c} Y\left(3\right)=-\frac{3}{16},\\ Z(3)=0; \end{array}\right.$
— for k=3: $\left\{\begin{array}{c} 4Y\left(4\right)=-\frac{1}{6},\\ 4Z(4)=0, \end{array}\right.$ hence we get $\left\{\begin{array}{c} Y\left(4\right)=-\frac{1}{24},\\ Z(4)=0,\;\;\mathrm{etc}. \end{array}\right.$

Limiting the *k* values to k=5 we get the approximate solution

$$y_{5}\left(x\right)=-\frac{1}{2}-\frac{3}{4}x-\frac{1}{2}x^{2}-\frac{3}{16}x^{3}-\frac{1}{24}x^{4}-\frac{1}{160}x^{5},\;\;z_{5}\left(x\right)=1.$$

**Figure 13:**
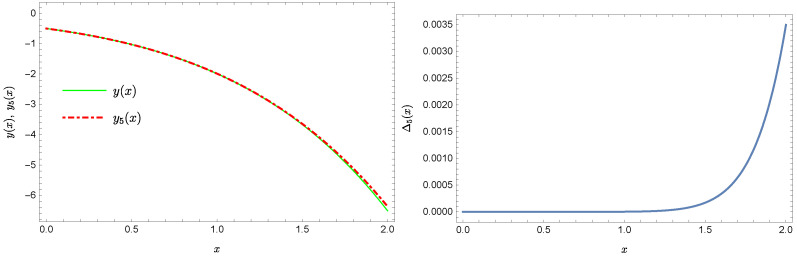
Exact solution and the approximate solution $y_{5}\left(x\right)$ together with the error Δ of this approximation.

**Figure 14:**
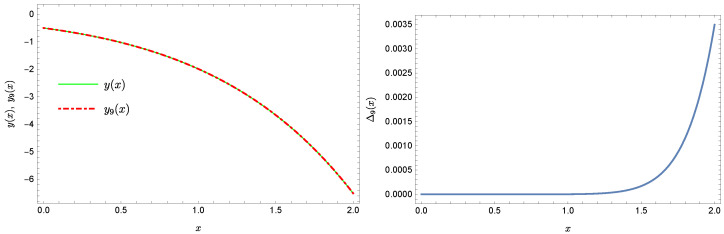
Exact solution and the approximate solution $y_{10}\left(x\right)$ together with the error Δ of this approximation.

Since function *z* is reconstructed exactly, Figure 13 presents only the comparison of the exact solution *y* and the approximate solution $y_{5}$ (the notation of figures is the same as in the previous examples) together with the graph of absolute error $\Delta_{5}\left(x\right)$ of this approximation. Next, Figure 14 shows the corresponding graphs for the approximate solution $y_{10}$.

Interesting is that we obtain in the successive steps the exact values of coefficients of the expansions of functions y(x) and z(x) into the Taylor series, which means that if only we would be able to discover this relation, we could obtain the exact solution of System (5).

## 5. Conclusions

This paper discusses the possibility of applying the Taylor differential transformation for solving the integro-differential equations with a retarded argument. The investigated equations are important, because we can find them in various mathematical, technical and engineering problems, and simultaneously, solving them is not an easy task. So, the contribution of the presented research is the development of an efficient technique for the solution of nonlinear equations which may arise in the area of up-growing interest, like the wireless sensor network and industrial internet of things. Examples, presented in this elaboration, show that the solution technique based on the Taylor differential transformation is efficient in case of equations of considered kind and its additional advantage is the simplicity of its application. The discussed method is well applicable when the sought function is expandable into the Taylor series in the considered region. In all cases, examined in the current paper and elsewhere, the satisfactory solution was possible to find always when this assumption was satisfied. In future we plan to compare the discussed method with some similar methods based on other transformations, like, for example, the Laplace transform. Moreover, we plan to apply DTM for solving some problems described by the equations with fractional derivative.

## Data Availability

Not applicable.
